# Pangenome of water caltrop reveals structural variations and asymmetric subgenome divergence after allopolyploidization

**DOI:** 10.1093/hr/uhad203

**Published:** 2023-10-13

**Authors:** Xinyi Zhang, Yang Chen, Lingyun Wang, Ye Yuan, Mingya Fang, Lin Shi, Ruisen Lu, Hans Peter Comes, Yazhen Ma, Yuanyuan Chen, Guizhou Huang, Yongfeng Zhou, Zhaisheng Zheng, Yingxiong Qiu

**Affiliations:** Systematic and Evolutionary Botany and Biodiversity Laboratory, College of Life Sciences, Zhejiang University, Hangzhou, 310058, Zhejiang, China; CAS Key Laboratory of Plant Germplasm Enhancement and Specialty Agriculture, Wuhan Botanical Garden, Chinese Academy of Sciences, Wuhan, 430074, Hubei, China; Systematic and Evolutionary Botany and Biodiversity Laboratory, College of Life Sciences, Zhejiang University, Hangzhou, 310058, Zhejiang, China; CAS Key Laboratory of Plant Germplasm Enhancement and Specialty Agriculture, Wuhan Botanical Garden, Chinese Academy of Sciences, Wuhan, 430074, Hubei, China; Provincial Key Laboratory of Characteristic Aquatic Vegetable Breeding and Cultivation, Jinhua Academy of Agricultural Sciences (Zhejiang Institute of Agricultural Machinery), Jinhua, 321000, Zhejiang, China; Jiaxing Academy of Agricultural Sciences, Jiaxing, 314016, Zhejiang, China; Provincial Key Laboratory of Characteristic Aquatic Vegetable Breeding and Cultivation, Jinhua Academy of Agricultural Sciences (Zhejiang Institute of Agricultural Machinery), Jinhua, 321000, Zhejiang, China; Provincial Key Laboratory of Characteristic Aquatic Vegetable Breeding and Cultivation, Jinhua Academy of Agricultural Sciences (Zhejiang Institute of Agricultural Machinery), Jinhua, 321000, Zhejiang, China; Institute of Botany, Jiangsu Province and Chinese Academy of Sciences, Nanjing 210014, Jiangsu, China; Department of Environment & Biodiversity, Salzburg University, Salzburg, 5020, Austria; CAS Key Laboratory of Plant Germplasm Enhancement and Specialty Agriculture, Wuhan Botanical Garden, Chinese Academy of Sciences, Wuhan, 430074, Hubei, China; CAS Key Laboratory of Plant Germplasm Enhancement and Specialty Agriculture, Wuhan Botanical Garden, Chinese Academy of Sciences, Wuhan, 430074, Hubei, China; State Key Laboratory of Tropical Crop Breeding, Shenzhen Branch, Guangdong Laboratory of Lingnan Modern Agriculture; Key Laboratory of Synthetic Biology, Ministry of Agriculture and Rural Affairs, Agricultural Genomics Institute at Shenzhen, Chinese Academy of Agricultural Sciences, Shenzhen 518124, Guangdong, China; State Key Laboratory of Tropical Crop Breeding, Shenzhen Branch, Guangdong Laboratory of Lingnan Modern Agriculture; Key Laboratory of Synthetic Biology, Ministry of Agriculture and Rural Affairs, Agricultural Genomics Institute at Shenzhen, Chinese Academy of Agricultural Sciences, Shenzhen 518124, Guangdong, China; Provincial Key Laboratory of Characteristic Aquatic Vegetable Breeding and Cultivation, Jinhua Academy of Agricultural Sciences (Zhejiang Institute of Agricultural Machinery), Jinhua, 321000, Zhejiang, China; CAS Key Laboratory of Plant Germplasm Enhancement and Specialty Agriculture, Wuhan Botanical Garden, Chinese Academy of Sciences, Wuhan, 430074, Hubei, China

## Abstract

Water caltrop (*Trapa* spp., Lythraceae) is a traditional but currently underutilized non-cereal crop. Here, we generated chromosome-level genome assemblies for the two diploid progenitors of allotetraploid *Trapa. natans* (4x, AABB), i.e., diploid *T. natans* (2x, AA) and *Trapa incisa* (2x, BB). In conjunction with four published (sub)genomes of *Trapa*, we used gene-based and graph-based pangenomic approaches and a pangenomic transposable element (TE) library to develop *Trapa* genomic resources. The pangenome displayed substantial gene-content variation with *dispensable* and *private* gene clusters occupying a large proportion (51.95%) of the total cluster sets in the six (sub)genomes. Genotyping of presence-absence variation (PAVs) identified 40 453 PAVs associated with 2570 genes specific to A- or B-lineages, of which 1428 were differentially expressed, and were enriched in organ development process, organic substance metabolic process and response to stimulus. Comparative genome analyses showed that the allotetraploid *T. natans* underwent asymmetric subgenome divergence, with the B-subgenome being more dominant than the A-subgenome. Multiple factors, including PAVs, asymmetrical amplification of TEs, homeologous exchanges (HEs), and homeolog expression divergence, together affected genome evolution after polyploidization. Overall, this study sheds lights on the genome architecture and evolution of *Trapa,* and facilitates its functional genomic studies and breeding program.

## Introduction

Underutilized or ‘neglected’ crops are mostly wild or semi-domesticated species that have been used for food, medicine or cultural practices (etc.) for centuries but are no longer widely used or commercialized as part of mainstream agriculture ([1]; see also [2–4]). Nevertheless, many underutilized crops possess a high content of micronutrients for mitigating malnutrition, and are often adapted to unique climatic and environmental conditions (e.g. [3]). Thus, they are not only important to local people, but can also have the potential to improve the resilience and sustainability of food production systems [4, 5].

The annual herbaceous and aquatic genus *Trapa* L. (Lythraceae), has traditionally been divided into two species, i.e., *Trapa natans* L. with diploid (2*n* = 2x = 48) and tetraploid (2*n* = 4x = 96) cytotypes, and diploid *Trapa incisa* Sieb. and Zucc. (2*n* = 2x = 48) [6, 7]. The fruits of *Trapa* spp*.*, also known as water caltrop, possess a high content of starch and were once an important food source, but are presently mostly underutilized [8]. Archaeological evidence suggests that water caltrop has been domesticated in the Yangtze River basin since the Neolithic period [9]. Based on the assembly of tetraploid *T. natans* and population genomics analyses of wild and cultivated accessions of *Trapa* in our previous study (Fig. S1) [10], we have found that tetraploid *T. natans* (AABB) is an allotetraploid hybrid between diploid *T. natans* (AA) and *T. incisa* (BB); the cultivated water caltrop was domesticated from diploid *T. natans* at *c*. 6300 (5600–13 900) yrs bp, and was subject to further artificial selection in historical times, during the Tang and Song Dynasties (618–1279 ad). In addition, recent whole-genome sequencing and genome analysis of diploid *T. natans* and *T. incisa* have uncovered abundant genomic variations between the two species [11].

Genomic structural variants (SVs, variants ≥ 50 bp) are important sources of functional variation, and can play important roles in domestication, adaptation and speciation [12–14]. With the exponentially rising amount of reference genomes, extensive SVs have been discovered even within species, implying that a single reference genome is not sufficient to infer the full species genetic diversity (reviewed in [15]). Thus, generating a species-representative genome or ‘pangenome’ is the method of choice for better capturing both structural and nucleotide diversity [16, 17]. Pangenomes have recently been generated for various crops, such as soybean [18], rice [19, 20], maize [21], and wheat [22]. All these studies have highlighted the essential role of presence-absence variations (PAVs) within a species in determining the genetic basis of agronomic traits. For instance, PAVs localized within *MYB* genes were identified as a potential cause underlying the variation of grain colour in sorghum [23]. However, until now, only a few pangenome studies have been performed on underutilized crops, such as sesame [24] or pigeon pea [25].

In this study, we generated two chromosome-level genome assemblies of diploid *T. natans* (2x, AA) and *T. incisa* (2x, BB). These two newly sequenced genomes, and three previously published genome of *Trapa* [10, 11], were employed to construct a gene-based pangenome of *Trapa*. Based on this pangenome, we defined the *core*/*dispensable*/*private* gene clusters. We also built a pan transposable element (TE) library, and compared the divergence of TEs among the two subgenomes of allotetraploid *T. natans* and the four (sub)genomes of its diploid progenitors. In addition, we generated a graph-based pangenome to genotype PAVs, and identified genes with PAVs that likely contributed to speciation and phenotypic divergence between diploid *T. natans* and *T. incisa.* Finally, we investigated the genomic variations during allopolyploidization and subgenome dominance in allotetraploid *T. natans*. Overall, our study will contribute to a better understanding of the genome evolution underlying the diversification and polyploidization of *Trapa*. This pangenome resource will facilitate further studies on the evolutionary and functional genomics of water caltrop.

## Results

### *De novo* genome assembly and annotation of diploid *T. natans* and *T. incisa*

To construct the pangenome representing the full range of genetic diversity of *Trapa*, we sequenced and assembled the genomes of a traditional cultivar of diploid *T. natans* (i.e., a cultivar called ‘Nahuling’ with no horns, hereinafter referred to as ‘*TnA_NL*’) and one sample of *T. incisa* from Heilongjiang River, China (hereinafter referred to as ‘*TiB_HR*’). By adopting a hybrid assembly approach, we used a combination of PacBio long reads, Illumina short reads, and a Hi-C chromatin interaction map (see details in Materials and methods). The assembled genome of the *TnA_NL* was 477.43 Mb with a contig N50 of 6.27 Mb, which was 89.3% of the estimated genome size (534.47 Mb) determined by *k*-mer analysis. The resulting genome of *TiB_HR* was 470.61 Mb with a contig N50 of 12.07 Mb, accounting for 93.4% of the estimated genome size (503.46 Mb) (Table 1; Figs S2 and S3, see online supplementary material). Both assembled genomes showed low heterozygosity (*TnA_NL*: 0.31%; *TiB_HR*: 0.07%). The chromosome-scale scaffolds were finally assembled based on Hi-C data. Approximately 99.7% (475.99 Mb) and 97.8% (460.48 Mb) of the assembled sequences were anchored onto the respective 24 pseudo-chromosomes of *TnA_NL* and *TiB_HR*, respectively (Table 1; Table S1 and Figs S4 and S5, see online supplementary material). Based on our analyses of Benchmarking Universal Single-Copy Orthologs (BUSCO), we identified 1614 universal single-copy genes and most of them could be fully annotated onto the genome assemblies of *TnA_NL* (1578; 97.77%) and *TiB_HR* (1571; 97.34%) (Table 1). Core Eukaryotic Genes Mapping Approach (CEGMA) analyses revealed that 236 (95.16%) and 235 (94.76%) of the 248 core eukaryotic genes were present in complete length in the respective genomes (Table S2, see online supplementary material). In addition, a very high proportion of Illumina short reads could be remapped to each assembled genome (*TnA_NL*: 98.70%; *TiB_HR*: 97.41%) (Table S3, see online supplementary material). In total, 32 457 and 34 940 protein-coding genes were predicted for *TnA_NL* and *TiB_HR*, with an average of 5.27 and 5.09 exons per gene, respectively (Table 1; Tables S4 and S5, see online supplementary material)*.*

**Table 1:** Summary of genome assembly and annotation of *Trapa*

**Genomic features**	**TiB_HR**	**TiB_YR** ^**a**^	**TnA_NL**	**TnA_WL** ^**a**^	**Tn_tetra** ^**b**^
Species	*T. incisa*	*T. incisa*	*T. natans*	*T. natans*	*T. natans*
Genome composition	BB	BB	AA	AA	AABB
Location/Cultivar	Heilongjiang River	Yangtze River	Nahuling	Wuling	
Assembly size (Mb)	470.61	463.97	477.43	479.9	1056.98
Number of scaffolds	298	208	33	194	2873
Number of contigs	298	262	249	325	3854
Contig N50 (Mb)	12.07	13.77	6.27	13.52	3.19
Scaffold N50 (Mb)	12.07	13.77	21.23	13.52	20.8
Genome in chromosomes	97.84%	98.14%	99.70%	98.01%	89.30%
Number of annotated genes	34 940	33 315	32 457	33 306	68 946
Average number of exons per gene	5.09	5.45	5.27	5.48	5.09
Complete BUSCO	98.80%	97.60%	98.00%	97.70%	97.80%

a Information of diploid *T. natans* and *T. incisa* is cited from [11]. ^b^Information of allotetraploid *T. natans* is cited from [10].

### Construction of the gene-based pangenome of *Trapa*

**Figure 1:**
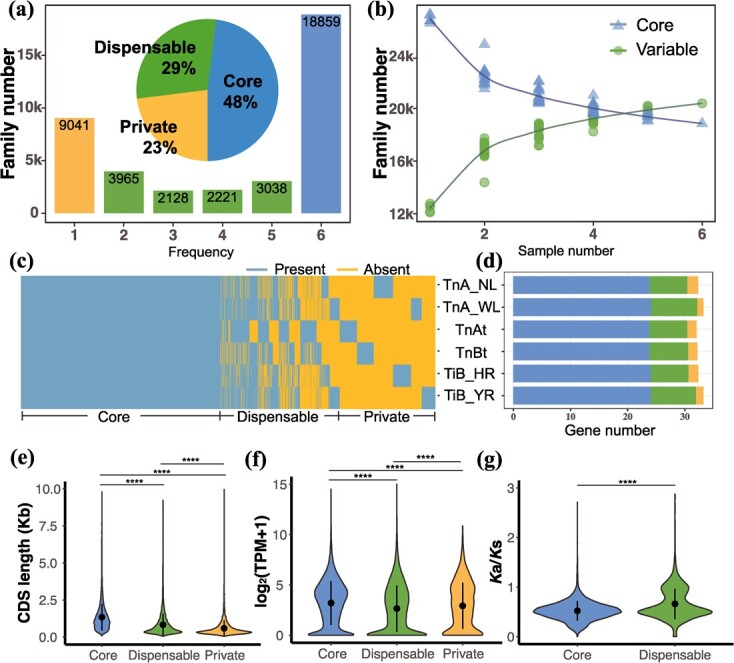
Composition and characteristics of the gene-based pangenome of *Trapa*. **(a)** Compositions of the gene-based pangenomes. The histogram shows the number of gene family clusters in the six (sub)genomes (diploid *T. natans*: *TnA_NL and TnA_WL*; diploid *T. incisa*: *TiB_HR* and *TiB_YR*; allotetraploid *T. natans*: *TnAt* vs. *TnBt*) with different frequencies. The pie chart shows the proportion of *core*, *dispensable*, and *private* gene family clusters. **(b)** Variation of variable (i.e., *dispesable* and *private* gene family clusters) and *core* gene family clusters with the number of water caltrop genomes increasing. **(c)** Presence and absence information of pan-gene family clusters in the six water caltrop (sub)genomes. **(d)** The number of classified genes in each (sub)genome. Summary of individual gene characteristics per gene cluster in terms of **(e)** CDS length, **(f)** expression level, and **(g)***K*a/*K*s values of each gene in core, dispensable, and private gene clusters. Asterisks denote statistical significance across three types of gene clusters.

Following the protocol of Lu *et al*. [10], we distinguished two subgenomes, termed ‘A-subgenome’ vs. ‘B-subgenome’, within the published genome of tetraploid *T. natans* [10]*.* In total, six assembled (sub)genomes, including two genomes of diploid *T. natans* (*TnA_NL* and *TnA_WL*) (this study), two genomes of *T. incisa* (*TiB_HR* and *TiB_YR*) [11], and two subgenomes of allotetraploid *T. natans* (*TnAt* and *TnBt*) [10], were used to construct a gene-based pangenome (Table 1). Sequence similarity analysis classified the predicted genes from the six (sub)genomes into 39 252 non-redundant gene clusters. Of these gene clusters, 18 859 (48.05%), 11 352 (28.92%) and 9041 (23.03%) were defined as *core* gene clusters, *dispensable* gene clusters, and *private* gene clusters, respectively (Fig. 1a and c). As shown in Fig. 1b, the number of *core* genes found in all genomes decreases as the number of genomes increases. The *dispensable* and *private* gene clusters occupied a large proportion (51.95%) of the total cluster sets in the six accessions. However, in individual accessions, the genes from the *dispensable* and *private* gene clusters averaged only 26.38% of the total genes (Fig. 1d). In addition, all six (sub)genomes contained a similar proportion of *core* (72.42–74.41%), *dispensable* (20.27–24.32%), and *private* (3.09–5.32%) genes (Fig. 1d).

The average lengths of the *dispensable* (832 bp) and *private* (590 bp) genes were significantly shorter than those of the *core* genes (1349 bp) (*P* < 2.2e-16, Wilcoxon rank-sum test) (Fig. 1e). Based on our RNA sequencing (RNA-seq), *core* genes [3.21 log_2_(TPM + 1)] had significantly higher expression levels than *dispensable* genes [2.65 log_2_(TPM + 1), *P* < 2.2e-16] and *private* genes [2.92 log_2_(TPM + 1), *P* = 8.3e-13, Wilcoxon rank-sum test] (Fig. 1f). Interestingly, a significantly higher ratio of non-synonymous/synonymous nucleotide substitutions (*K*a/*K*s) was observed in *dispensable* as compared to *core* genes (1.71 vs. 0.54, *P* < 2.2e-16, Wilcoxon rank-sum test) (Fig. 1g). Gene Ontology (GO) enrichment analysis indicated that *core* genes were significantly enriched for essential functions, including photoperiodism (e.g., GO:0048573; GO:0009658), meristem (e.g., GO:0000725; GO:0061982), and several terms related to chromosome organization (e.g., GO:0051276; GO:0098813) (Fig. S6 and Table S6, see online supplementary material). In contrast, *dispensable* genes were significantly enriched in biological processes related to root development (e.g., GO:0048527; GO:0080022), biosynthesis of secondary metabolites (e.g., GO:0009787; GO:0009699), and response to abiotic stress (GO:0071456; GO:0009416) (Fig. S7 and Table S7, see online supplementary material).

### Comparison of TEs between allotetraploid *T. natans,* diploid *T. natans*, and *T. incisa*

**Figure 2:**
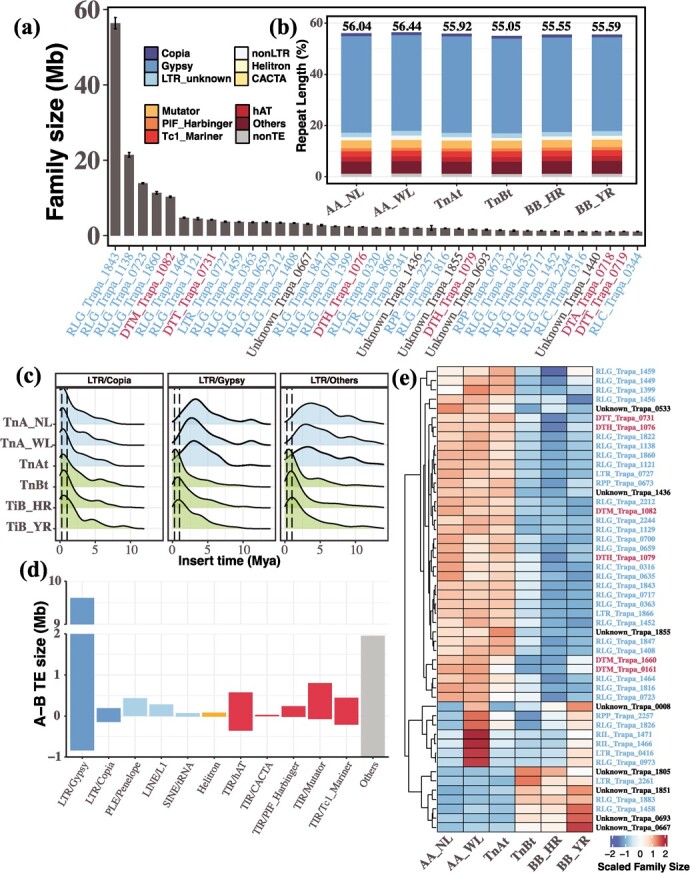
The landscape and insertion times of transposable elements (TEs) of *Trapa*. **(a)** Mean size of the 39 largest TE families (>1 Mb) across the six (sub)genomes (diploid *T. natans*: *TnA_NL* and *TnA_WL*; diploid *T. incisa*: *TiB_HR* and *TiB_YR*; allotetraploid *T. natans*: *TnAt* snd *TnBt*). The x-axis indicates the names of the TE families (blue: retrotransposons; red: DNA transposons). The error bars denote the standard deviation among the six (sub)genomes of *Trapa*. **(b)** Length (%) of repetitive elements per (sub)genome, as inferred by panEDTA annotation. **(c)** Estimated insertion times (in million years ago, Mya) of full-length long terminal repeat (FL-LTR) retrotransposons (*Copia*, *Gypsy*, and others) for each (sub)genome. Density distributions represent the A- (*TnA_NL*, *TnA_WL*, and *TnAt*) and B- (*TiB_HR*, *TiB_YR*, and *TnBt*) lineages, respectively. The two dashed lines represent the inferred times of the allotetraploidization (left, *c*. 0.27 Mya) and the divergence between diploid *T. incisa* and *T. natans* (right, *c*. 1 Mya). **(d)** Size differences in major TE families between the A- and B-lineages. Positive values represent families that are larger in the A-lineage than in the B-lineage, while negative values represent those that are larger in the B-lineage than in the A-lineage. **(e)** Heatmap of the scaled sizes of the 50 most different TE families between A- and B-lineages. Column names in blue vs. red indicate retrotransposons vs. DNA transposons.

TEs are among the most variable parts of the genome and may be important drivers of rapid adaptation and species divergence (e.g. [26, 27]). To examine the differences in TE distribution between species, we constructed a pan-TE library of the water caltrop pangenome, including a total of 1616 TE families, and re-annotated the six (sub)genomes (Fig. 2a; Table S8, see online supplementary material). As a result, TE and non-TE repeats accounted for 55.05% to 56.44% of the (sub)genomes (average: 55.77%), whereby long terminal repeat retrotransposons (LTR-RTs) were predominant (average: 40.16%; Fig. 2b; Table S9, see online supplementary material). The two main classes of of LTR-RTs were *Gypsy* (RLG) and *Copia* (RLC) elements, making up 37.20% and 1.17% of the (sub)genomes, respectively. For each (sub)genome, the size of TE families varied from a single element to 30 665 elements, with the 10 largest families accounting for an average of 52.39% of all TEs (Fig. 2a; Table S8, see online supplementary material).

The vast majority of TE families (1564 out of 1616, 96.78%) were consistently observed across the six (sub)genomes, but their sizes varied widely. For the A (i.e., *TnA_NL, TnA_WL* and *TnAt*) and B (i.e., *TiB_HR, TiB_YR* and *TnBt*) lineages, 164 TE families differed by at least 10 kb in size, of which 127 families (77.44%, including 57 *Gypsy* families) were larger in A-lineage as compared to B-lineage (Fig. 2d and e; Table S8, see online supplementary material). Burst time analysis of intact LTR-RTs revealed that *Copia* families in both A- and B-lineages experienced a recent burst (or expansion) at *c*. 0.29 million years ago (Mya) (Fig. 2c), possibly coinciding with a recent allopolyploidization event (*c*. 0.27 Mya) [10]. However, the expansion of the *Gypsy* families occurred much earlier in the A-lineage (*c*. 3.04 Mya) than in the B-lineage (*c*. 0.48 Mya) (Fig. 2c).

### Construction of the graph-based pangenome

**Figure 3:**
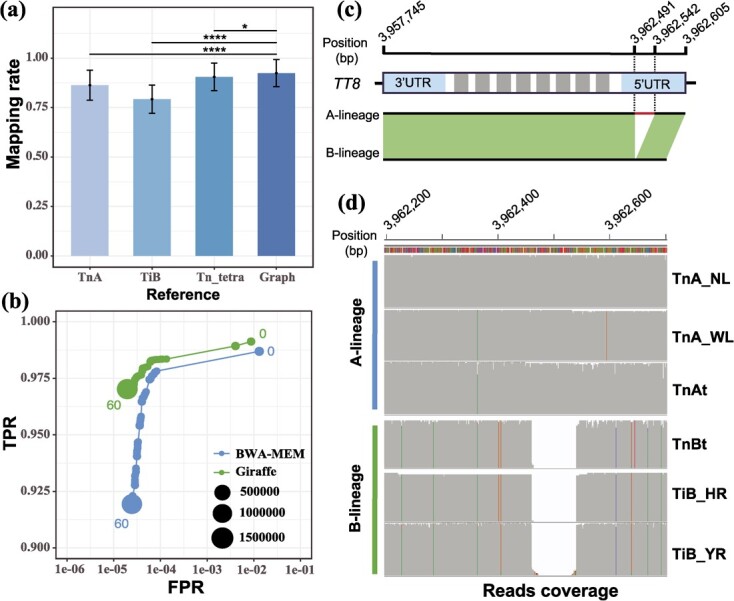
Comparison of mapping accuracy between graph and linear genomes. **(a)** The genome mapping rate of re-sequenced individuals with respect to three reference genomes of diploid *T. natans* (*TnA*), diploid *T. incisa* (*TiB*), and allotetraploid *T. natans* (*Tn_tetra*) and the graph-based pangenome of *Trapa*, respectively. **(b)** Receiver operating characteristic (ROC) graph illustrating true-positive vs. false-positive rates (TPRs vs. FPRs) at different mapping quality thresholds (*i* = 0–60) for graph-based and linear genome mapping approaches, respectively. The size of each circle is proportional to the log-scaled number of reads with the respective mapping quality. **(c)** An 80-bp deletion is located within the 5’UTR of the *TT8* gene of B lineage. The blue and grey boxs represent UTRs and exons, respectively. **(d)** Validation of the deletion based on the mapping of Pac Bio long reads onto the genome assembly of *TnA_NL*.

We constructed a graph-based pangenome for *Trapa* using the assembled (sub)genomes. To this aim, we chose the genome of diploid *T. natans* (*TnA*_*NL*) as the ‘backbone’, and identified insertions and deletions (≥ 50 bp) from the six (sub)genomes with long reads. Subsequently, 211 598 non-redundant PAVs were integrated into a variation graph. The resulting graph-based pangenome of *Trapa* spanned 558.12 Mb, of which about 80.69 Mb were absent from the *TnA* genome. To validate the quality of the graph-based pangenome, the paired-end short reads of 57 individuals, including allotetraploid *T. natans* and diploid *T. natans*/*T. incisa* from our previous study [10], were mapped on the graph and linear genomes, respectively. On average, 92.42% of the reads were properly mapped on the graph, which was much higher than that obtained from linear-genome mapping (79.18–90.52%; Fig. 3a). Moreover, our simulation studies showed that the graph-based mapping had either higher precision or higher recall relative to the linear mapping (Fig. 3b; Fig. S8, see online supplementary material).

As PAVs can largely contribute to the observed phenotypic variations (reviewed in [28]), we further genotyped the PAVs among six (sub)genomes based on the water caltrop graph-based pangenome. After filtering, 156 616 PAVs were retained, of which 40 453 (25.90%) were differentiated between A- and B-lineages. A total of 2570 genes were found to contain inter-lineage PAVs. These genes were mainly enriched in GO terms involving three biological processes/physiological pathways, i.e., organ development process, organic substance metabolic process and response to stimulus (Table S10, see online supplementary material). Not unexpectedly, a subset of these genes with PAVs were found to play a crucial role in phenotypic divergence and reproductive isolation between two diploids *TnA* and *TiB* (Fig. 3c and d; Fig. S1 and Table S11, see online supplementary material). For example, 102 genes (e.g., *TT8*, *CRY2*, *GED1*, *AGL61*) were involved in the developmental processes of flower, seed, and fruit, of which *PID* and *CRA1* play a role in asymmetric cotyledon development; 21 genes (e.g., *ECT2*, *PSY1R*, *SIZ1*, *ORE15*) were identified as potentially related to cell proliferation and expansion during plant organogenesis, which could contribute to the difference in plant size between *TnA* and *TiB*. In addition, 12 genes (e.g., *SS3*, *SS4*, *SPS2F*, *SBE2.1*) were found to be associated with the biosynthesis of sucrose/starch (Table S11, see online supplementary material). In addition, based on gene expression profile differences in four tissues of *TnA_NL* and *TiB_HR*, including flower bud (FB), fertilized flower (FF), juvenile fruit (JF), and leaf (L), 1428 genes were found to differentially express in at least one tissue (Table S12, see online supplementary material). Permutation test indicated that the number of differentially expressed genes (DEGs) was significantly higher than expected by chance (*P* < 2e-16).

### Evolutionary trajectory of genes affected by PAVs during polyploidization

**Figure 4:**
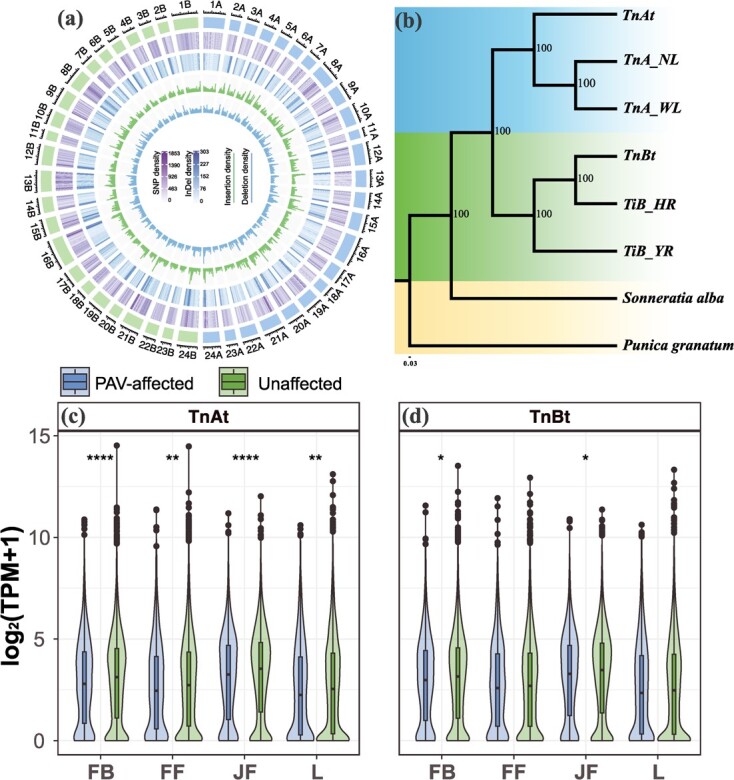
Phylogenetic relationships of *Trapa* accessions and distribution of genomic variations between subgenomes of allotetraploid *T. natans* and its progenitor diploid genomes. **(a)** Circos plot of the 24 chromosomes of *TnAt* (CHR_1At–24At) and *TnBt* (CHR_1Bt–24Bt), respectively, shows the density distributions of genomic variations compared to their presumed progenitor diploid genomes (*TnA_NL* and *TiB_HR*). SNPs, InDels, PAVs are included in this plot. **(b)** The maximum likelihood tree of *Trapa* based on 4150 single-copy orthologous genes. *Sonneratia alba* and *Punica granatum* were used as outgroups [29, 30]. **(c)** and **(d)** Violin plot of expression levels of PAV-affected genes in **(c)** the *TnAt* and **(d)***TnBt* subgenomes for each of four tissues (FB: flower bud; FF: fertilized flower; JF: juvenile fruit; and L:leaf).

To estimate the genomic variation during allopolyploidization in *Trapa*, we performed pairwise whole-genome alignments of each subgenome and genomes of its presumed diploid progenitors. On average 1 569 241 SNPs, 271 920 small InDels (<50 bp), 6260 deletions (≥50 bp), and 7094 insertions (≥50 bp) were identified in the A-subgenome (*TnAt*); 3 327 898 SNPs, 433 964 InDels, 12 300 deletions, and 14 213 insertions were detected in the B-subgenome (*TnBt*) (Fig. 4a; Table S13, see online supplementary material). Besides, we found much more variations between *TiB_YR* vs. *TnBt* as compared to *TiB_HR* vs. *TnBt*, consistent with the phylogenetic tree of *Trapa* (Fig. 4b; Table S13, see online supplementary material). Thus, we chose the reference genome of *TnA_NL* and *TiB_HR* as representatives for the following comparative analyses of subgenomes.

We further defined homeologous genes with PAVs occurring within 2 kb upstream and downstream of protein-coding genes as potentially *cis*-regulated genes. Of the 14 212 single-copy orthologous genes shared among the four (sub)genomes, we identified 3234 homeologous pairs with PAVs. Of them, 1623 genes were shared between the two subgenomes of allotetraploid *T. natans* (*TnAt* and *TnBt*), while 785 and 826 were specific to *TnAt* and *TnBt*, respectively. Those potentially PAV-affected genes in both subgenomes related to allopolyploidization had significantly higher *K*a/*K*s ratios than those for unaffected genes (both *K*a/*K*s values <0.5; P = 0.001 for *TnAt*, *P* = 3.32-6e for *TnBt*, Wilcoxon rank-sum test) (Fig. S9, see online supplementary material), indicating that these PAV-affected genes underwent relaxed purifying selection. We further evaluated the effects of PAVs on levels of gene expression in four tissues, i.e., flower bud (FB), fertilized flower (FF), juvenile fruit (JF), and leaf (L). The average expression levels of PAV-affected genes were significantly lower than those of the remaining genes in all four tissues of *TnAt*, especially for flower bud and juvenile fruit (Fig. 4c and d). However, when compared with the unaffected genes, the PAV-affected genes had a significantly lower expression level only in the flower bud and juvenile fruit of *TnBt*. This suggests that PAVs could have a greater impact on homologous gene expression in *TnAt* than in *TnBt*.

### Homeologous exchanges and homeolog expression pattern

**Figure 5:**
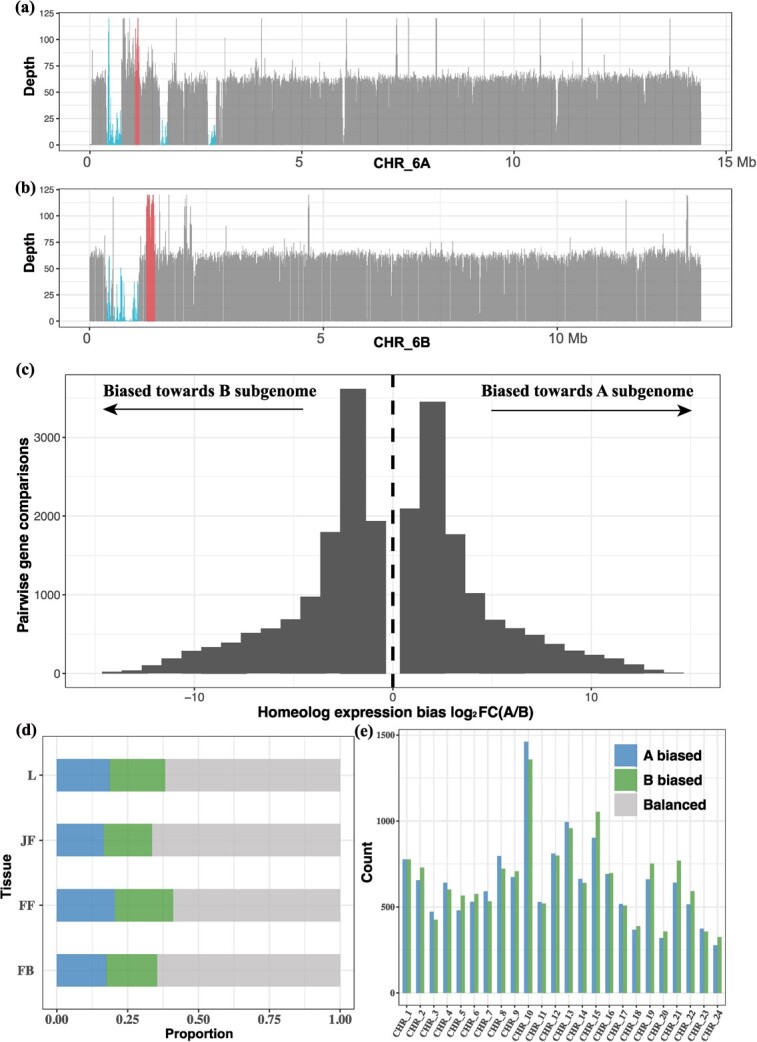
Homeologous exchange (HE) regions and homeologous expression bias (HBE) in the two subgenomes (*TnAt* vs. *TnBt*) of allotetraploid *Trapa natans* (2*n* = 4x = 96). ****(a)** and **(b)**** HE regions, as exemplified for one chromosome each of the *TnAt* and *TnBt* subgenomes. Coverage depth was obtained for CHR_6At **(a)** and CHR_6Bt chromosomes **(b)** after mapping Illumina sequence reads from allotetraploid *T. natans* on the diploid *T. natans* and *T. incisa* genome assemblies concatenated together. The regions with red lines represent windows whose coverage depth was 1.5 times greater than the whole-genome average depth, while the blue lines show deletions with low or no coverage. **(c)** Histograms of genome-wide expression of syntenic homeologous genes among the *TnAt* and *TnBt* subgenomes. ****(d)** and (e)** Pairwise comparisons between syntenic gene pairs among four tissues (FB: flower bud; FF,: fertilized flower; JF: juvenile fruit; L: leaf) **(d)** and among the 24 chromosome pairs (**e**).

Homeologous exchange (HE) between the constituent subgenomes is a frequent type of structural variant in polyploids [31]. In this study, we identified 61 HEs in allotetraploid *T. natans*, including 35 from *TnBt* to *TnAt* (5.08 Mb) and 26 from *TnAt* to *TnBt* (3.67 Mb) (Fig. 5a and b; Tables S14–S16, see online supplementary material). Functional annotation of genes within HEs revealed that these genes were enriched in organ development [e.g., trichome morphogenesis (GO:0010090), leaf senescence (GO:0010150), and leaf development (GO:0048366)] and stress response processes [e.g., response to light intensity (GO:0009642), cellular response to oxygen-containing compound (GO:1901701), and cellular response to endogenous stimulus (GO:0071495)] (*P* < 0.05) (for details, Tables S14–S16, see online supplementary material), suggesting a role of asymmetric HEs in biological function.

Based on the expression profiles of the four tissues described above (flower bud, fertilized flower, juvenile fruit, and leaf), we tested for signatures of homeolog expression bias (HEB) in allotetraploid *T. natans*. Of the 24 966 syntenic gene pairs between the *TnAt* and *TnBt* subgenomes, 10 626 and 2241 displayed HEB in at least one tissue and all sampled tissues, respectively (Table S17, see online supplementary material). Based on pairwise comparisons between syntenic gene pairs, there was a slight bias in expression level towards the B-subgenome across all four tissues and most chromosome pairs (*P* = 0.022, Wilcoxon rank-sum test) (Fig. 5c–e; Fig. S10, see online supplementary material). At the chromosome level, a total of 13 pairs of chromosomes showed significant HEB (*P* < 0.05, Wilcoxon rank-sum test), and eight of those showed a bias in expression level towards the B-subgenome (Fig. 5e).

## Discussion

Considerable efforts are essential in the exploitation of sustainable food supply to meet the upcoming food production challenge [4, 21]. The cultivated diploid *Trapa natans*, containing about 67.5% high-quality starch of the fruit’s dry weight [32, 33], is a potential secure food source, especially in wasteland regions [8]. Even though the previously published reference genomes of allopolyploid *T. natans*, diploid *T. natans* and *T. incisa* provided strong support for its allotetraploid origin and domestication of diploid *T. natans* [10, 11], it does not capture fully the genetic variability within water caltrop.

In this study, we have constructed a gene-based pangenome dataset for *Trapa*. Based on this, the proportion of *core* gene clusters predicted in *Trapa* (48.05%) proved to be higher than those of soybean (35.87%, [18]), rice (30.58%, [19]), and sorghum (36%, [23]). This might be due to a combination of its relatively recent speciation and the limited size of genome assemblies. Nevertheless, the constructed *Trapa* pangenome exhibited extensive variation in gene content, as indicated by the prediction of 28.92% *dispensable* and 23.03% *private* gene clusters within the six (sub)genomes. Based on GO enrichment analyses, the *core* genes were mainly comprised of conserved and housekeeping genes (Fig. 1c–e; Fig. S6, see online supplementary material), while these *dispensable* genes were primarily related to biological processes related to organ development, metabolism, and biotic and abiotic stress response (Fig. S7, see online supplementary material). This implies that *dispensable* genes might have a crucial role in phenotypic variation and adaptation to abiotic/biotic stresses of water caltrop. Similar patterns are also found in the pangenomes of other crops, including rice [19], *Brassica* species [34, 35] sesame [24], soybean [18], and pigeon pea [25]. Interestingly, although we observed that *dispensable* genes had relatively lower expression level compared with *core* genes (Fig. 1f), their significantly higher *K*a/*K*s values (Fig. 1g) suggest that *dispensable* genes are evolving faster due to relaxed functional constraints, likely promoting speciation and phenotypic divergence (Fig. S7, see online supplementary material).

By not only focusing on SNPs and small InDels, many recent studies have also found that structural variations (SVs) play a major role in species diversification and phenotype variation in both plants and animals [14, 36, 37]. In our study, when compared to *T. incisa* (BB), diploid *T. natans* (AA) has stouter stems with larger fruits (seeds), leaves, and flowers and more vigorous roots (Fig. S1, see online supplementary material) [38]. To precisely identify the potential PAVs that may contribute to reproductive isolation and phenotypic divergence between *T. incisa* and diploid *T. natans*, we have constructed a graph-based pangenome based on 211 598 non-redundant PAVs from the six (sub)genomes. Consistent with patterns observed in recent studies on humans [39], yeasts [39], cows [40], and tomatoes [41], both the mapping rate and mapping accuracy of our graph-based pangenome of *Trapa* are much higher than that of the corresponding linear genome (Fig. 3a and b; Fig. S8, see online supplementary material). As expected, 2570 genes with essential functions were associated with PAVs specific to A- or B-lineages. For example, an 80-bp deletion within 5’UTR of *TT8* was found to be specific to B-lineage (Fig. 3c and d). *TT8*, a bHLH transcription factor, has been proven to affect post-zygotic reproductive barrier in *Arabidopsis thaliana* [42]. It was also differentially expressed between the two lineages among four tissues of *Trapa* (Table S12, see online supplementary material). Besides, some genes under positive selection in cultivated water caltrop (such as *PID* and *CRA1* [10]) were also found to be associated with PAVs (Table S11, see online supplementary material). This suggests that these genes are likely related to speciation and domestication. Clearly, however, in addition to the PAV information provided herein, further research is required to complement the graph-based pangenome of *Trapa* with other types of genomic SVs (e.g., copy number variations, inversions, and chromosomal rearrangements) as well as single nucleotide polymorphisms (SNPs) and small InDels, etc. [39].

Our findings have also revealed that the B-subgenome (*TnBt*) is the dominant one in allotetraploid *T. natans* based on inferences in four important respects. Firstly, when compared to *TnBt*, *TnAt* subgenome lost more genes but retained more TEs during polyploidization (Fig. 2a; Tables S9 and S18, see online supplementary material), suggesting this TE-rich subgenome may experience more gene losses, and eventually become a recessive subgenome due to ongoing gene loss [43]. Similar patterns have been observed in the genome of tetraploid broomcorn millet (*Panicum miliaceum*), one of the earliest domesticated crops [44]. Besides, PAVs were found to have a greater impact on homologous gene expression in *TnAt* than in *TnBt* (Fig. 4c and d). Further, partitioning of homeolog gene expression is largely established in allotetraploid *T. natans* with the presence of slight bias towards the B-subgenome across four tissues and most chromosome pairs (Fig. 5 c–e). Such gene expression variation between the two subgenomes may contribute to the increased complexity of regulatory networks after allopolyploidization (see also [43]). Finally, homeologous exchanges from *TnBt* to *TnAt* were found to be more frequent than the reverse (Table S14, see online supplementary material). These findings indicate that multiple factors, including PAVs, asymmetrical amplification of Tes, Hes, and homeolog expression divergence, together affect a route for genome evolution after polyploidization. PAV-affected genes in both subgenomes were found to be under natural selection, and some genes related to Hes were enriched in organ development and stress response processes. These genes might have contributed to the vigour and broad adaptation of allotetraploid *T. natans* (Tables S14–S16, see online supplementary material). In summary, the pangenome of Trapa affords a platform for a thorough exploration of genomic variation of Trapa species, thereby promoting a better understanding of the evolutionary and functional genomics of this currently underutilized crop variation.

## Materials and methods

### Plant materials

For genome sequencing, the plant sample of diploid *T. incisa* was collected from Xingkai Lake National Nature Reserve (132.32°E, 45.37°N), and that of diploid *T. natans* was collected at Jiaxing Academy of Agricultural Science (120.69°E, 30.86°N). For genome annotation of *T. incisa*, both Illumina short-read and PacBio long-read RNA sequencing were performed. The long-read transcriptome data were derived from the evenly mixed sample of six tissues, while short-read transcriptome data were generated from different tissues separately (Table S19, see online supplementary material). For genome annotation of *T. natans*, previously released short-read transcriptome sequencing data (SRR14597430–SRR14597415, [10]) separately derived from different tissues were used (Table S19, see online supplementary material).

### Genome assembly and quality assessment

Both genomes were assembled using a hybrid strategy that combined PacBio long reads, Illumina short reads, and a Hi-C chromatin interaction map. After quality controlling, 23.57 Gb of PacBio HiFi data, 55.73 Gb of Illumina data, and 40 Gb of Hi-C data (Table S20, see online supplementary material) were used for *de novo* genome assembly of *T. incisa*.Firstly, basing on *k*-mer frequency distribution analysis, Illumina short reads were used to estimate genome size and heterozygosity of this individual with jellyfish v.2.3.0 [45] and genomescope2 [46]. Then, PacBio HiFi reads were subjected to draft assembly with default parameters using hifiasm v.0.16.0 [47]. Finally, the Hi-C clean reads were aligned to the draft assembly with bwa-mem [48]. Allhic v.0.9.8 [49] was applied to perform genome assembly at the chromosome level using the corrected contigs. Juicebox tool v.2.12 [50] was applied to adjust chromosome construction manually. Benchmarking Universal Single-Copy Orthologs (busco v.4.0.5) [51] and Core Eukaryotic Genes Mapping Approach (cegma v.2.5) [52] were applied to evaluate the completeness of the genome assembly. In addition, Illumina reads were aligned to the reference genome to assess the mapping rate.

For diploid *T. natans*, 127 Gb of PacBio Continuous Long Reads (CLR) data, 33.7 Gb of Illumina data, and 40 Gb of Hi-C data were used for *de novo* genome assembly (Table S20, see online supplementary material). The clean reads were subjected to self-correction, trimming, and assembly using canu v.2.2 [53]. Afterwards, error-corrected contigs were assessed and anchored onto chromosomes, using the same pipeline as mentioned above.

### Annotations of transposable elements (TEs) and gene models

To predict the TEs in *Trapa* genomes, we first constructed a *de novo* TE library for each of the six (sub)genomes using the exstensive*de*-*novo*te annotator (edta v.2.1 [27, 54] with parameters ‘--overwrite 1 --sensitive 1 --anno 1 --evaluate 0’. Classifications of these TE libraries were refined using the script deepte.py with the predefined plant model [55]. Then, the script panedta.sh [27] was performed to generate the pan-TE library by eliminating low-copy, incomplete, and redundant sequences across individual TE libraries. Finally, this filtered pan-TE library was used to re-annotate all genomes with consistent TE family IDs, including both structurally intact and fragmented TEs.

Prediction of protein-coding genes of diploid *T. natans* and *T. incisa* was performed using the repeat-masked genome with three distinct approaches, i.e., *ab initio* gene prediction, homology-based prediction, and transcriptome-assisted annotation. For the *ab initio* gene prediction, augustus v.3.2.3 [56], glimmerhmm v.3.0.4 [57], genscan v.1.0 [58], snap v.2013.11.29 [59], and geneid v.1.4.4 [60] were applied. For the homology-based gene prediction, the protein-coding sequences from allotetraploid *T. natans* (PRJNA725399 [10]), *Corymbia citriodora* (PRJNA234431 [61]), *Eucalyptus grandis* (AUSX00000000 [62]), and *Punica granatum* (PRJNA355913 [63]) were mapped to the assembled genomes using tblastn v.2.2.26 (*E*-value ≤1e-5 [64]) to obtain high-quality protein structures. To perform transcripts-assistant annotation, PacBio ISO-seq and Illumina short-read RNA-seq data were used for *T. incisa* (see details in Table S19, see online supplementary material). Smrtlink v.11.1 (https://www.pacb.com/support/software-downloads/) and isoseq v.3 (https://github.com/PacificBiosciences/IsoSeq) were used to extract isoform sequences and process the polished consensus sequences. A total of 54.97 Gb PacBio ISO-Seq reads were directly mapped to the genome of *T. incisa* by gmap v.2021-12-17 [65]. The short-read RNA-seq data were aligned to the genome sequence with tophat v.2.0.11 [66]. Subsequently, the mapped reads were assembled into longer transcripts using cufflinks v.2.2.1 [67]. The transcripts from all tissues were merged and subjected to transdecoder in pasa v2.4.1 [68] to predict and filter protein-coding sequencing. Only complete transcripts were retained for further analysis. For the diploid *T. natans*, only previously released short-read transcriptome data were used to perform transcriptome-assisted annotation with the same pipeline as mentioned above (Table S19, see online supplementary material). All genes predicted by the above methods were integrated into a non-redundant gene set using evidencemodeler (evm) v.1.1.1 [68]. The EVM-predicted genes were further updated with pasa v.2.4.1 [68] to predict the untranslated regions and alternative splicings. The resulting protein models were functionally annotated according to the best matches with proteins deposited in go, kegg, swiss-prot, trembl and a non-redundant protein database using blastp (*E*-value = 1e-5).

### Construction of the gene-based pangenome of *Trapa*

To identify the *core*/*dispensable*/*private* gene sets, we clustered gene families using orthofinder v.2.2.7 [69]. Firstly, the genes containing coding sequence (CDS) with 100% similarity to other genes were removed using the cd-hit-est implemented in cd-hit v.4.8.1 [70] toolkit for each accession with parameters ‘–c 1 –aS 1’. Protein sequences of the remaining genes were then subjected to homologous searching by dimand balstp v.2.0.11.149 [71], with an *E*-value cutoff of 1e−5. Based on the results of this latter search, orthofinder was used for gene family clustering, with parameters ‘percentMatchCutoff = 50’ and ‘-I 1.5’. Gene clusters shared among all six accessions were defined as *core* gene clusters, while the gene clusters missed in more than one accessions were defined as *dispensable* gene clusters, and those only existed in single accession were defined as *private* gene clusters.

kaks_calculator v.2.0 [72] and the parallel tool paraat v.2.0 [73] were used to calculate *K*a/*K*s ratio to estimate the selection pressure acting on *core*/*dispensabl*e/*private* gene clusters. In addition, we performed GO enrichment analysis for each cluster at GOC website (http://geneontology.org/) under default parameters.

### Construction of the graph-based pangenome of *Trapa*

To construct a graph-based genome for the genus *Trapa*, we selected the *TnA* genome of diploid *T. natans* as the ‘backbone’, as it showed the highest contiguity and completeness among the four (sub)genomes. In a next step, the PacBio long reads of the four sub(genomes) were mapped onto the *TnA* genome using minimap2 v.2.24 [74]. PAVs were called using cutesv v.1.0.13 [75] with the suggested parameters. All PAVs across the four (sub)genomes were merged according to instructions provided on GitHub (https://github.com/vgteam/giraffe-sv-paper/blob/master/scripts/sv). Finally, the final graph-based genome of *Trapa* was constructed using the variation graph (vg v.1.38.0) toolkit pipeline [39].

Mapping accuracy of graph-based genome was assessed following the pipeline of human research (https://github.com/vgteam/giraffe-svpaper/blob/master/scripts/read_simulation/). Firstly, one million read pairs were simulated and mapped to the graph and linear genomes by vg giraffe [39] and bwa-mem [48], respectively. Subsequently, we calculated the cumulative true-positive rate (TPR) and false-positive rate (FPR) at different mapping quality thresholds to assess mapping sensitivity and specificity. The results were finally visualized as the receiver operating characteristic (ROC) curve.

### Assessment of genomic variation between diploids and subgenomes

The subgenomes of allotetraploid *T. natans*, respectively, were aligned to their progenitor diploid genomes using the nucmer program implemented in the mummer toolkit v.4.0.0 [76] with parameters ‘-maxmatch -c 100 -l 50’. The alignment results were filtered using the delta-filter program of mummer with only one-to-one alignment blocks retained using parameters ‘-1 -l 1000’. In addition, SNPs and InDels (<50 bp) were identified using show-snps (with potion **‘**-clrt**’**) from mummer v.4.0.0 [76], and the cutesv pipeline (see above) was used to identify large insertions/deletions ($\ge$50 bp).

### Gene expression analysis and identification of homeolog expression bias (HEB)

Transcriptome sequencing encompassed the use of three biological replicates of the flower buds (FB), fertilized flowers (FF), juvenile fruits (JF), and leaves (L) of diploid *T. natans*, allotetraploid *T. natans*, and *T. incisa*. Clean reads of RNA-seq were aligned to the allotetraploid *T. natans* genome, using star v.2.7.9a [77]. Only uniquely mapped reads were retained and used for counting reads of the annotated genes with featurecounts v.2.0.3 [78].

HEB was identified across all 1:1 homeologous gene pairs using deseq2 v.4.2 [79]. Comparisons were made between the two homeologous gene pairs for each tissue. We performed library size normalization for gene counts with the ‘Relative Log Expression’ normalization (RLE) method, as implemented in the deseq2 package. Log 2 fold change of a given gene was calculated using the Built-in Wald test. The genes with false discovery rate (FDR) corrected *P*-value <0.05 were considered significantly biased. DEGs between diploid *T. natans* and *T. incisa* were identified using deseq2 v.4.2.

### Identify HE regions between two subgenomes

Segmental HE regions between the two subgenomes of allotetraploid *T. natans* were identified according to the method of ‘assessment of read depth’ as successfully applied in the ‘*Brassica napus* genome project’ [31]. Briefly, Illumina paired-end reads of allotetraploid *T. natans* were mapped to diploid *T. natans* and *T. incisa* concatenated together. The average sequencing depth was calculated based on 10 kb non-overlapping window using bedtools v.2.30.0 [80]. Windows with read depth 1.5 times being greater than the average mapping depth of the whole genome were considered as candidate duplications generated by HEs. The windows with low or no coverage were regarded as deletions. Moreover, if adjacent windows had a depth above the set threshold and were within 50 kb (five windows), they were linked together. Only regions that extended beyond 80 kb were considered as probable HEs (see also [31]).

## Supplementary Material

Supplementary_FigureS1-S10_uhad203

Supplementary_TableS1-11_uhad203

Supplementary_TableS12_uhad203

Supplementary_TableS13-20_uhad203

## Data Availability

The whole genome sequencing data for diploid *T. natans* have been deposited under NCBI BioProject PRJNA932942 and GSA BioProject PRJCA016421. The whole genome sequencing data for diploid *T. incisa* have been deposited under NCBI BioProject PRJNA933001 and GSA BioProject PRJCA016421. The transcriptome sequencing data of diploid *T. incisa* and allotetraploid *T. natans* have been deposited under NCBI BioProject PRJNA941110 and PRJNA731291, respectively. The re-sequencing (57 accessions) and transcriptome sequencing data of diploid *Trapa natans* for genome annotation were obtained from the NCBI BioProject PRJNA725399 of Lu *et al.* [10].
