# Supplementary material for: Pangenome of water caltrop reveals structural variations and asymmetric subgenome divergence after allopolyploidization
Source: Hortic Res. 2023 Oct 13;10(11):uhad203. doi: 10.1093/hr/uhad203 (PMC10689057; doi:10.1093/hr/uhad203)
Supplement: Supplementary_FigureS1-S10_uhad203 [file supplementary_figures1-s10_uhad203.docx]

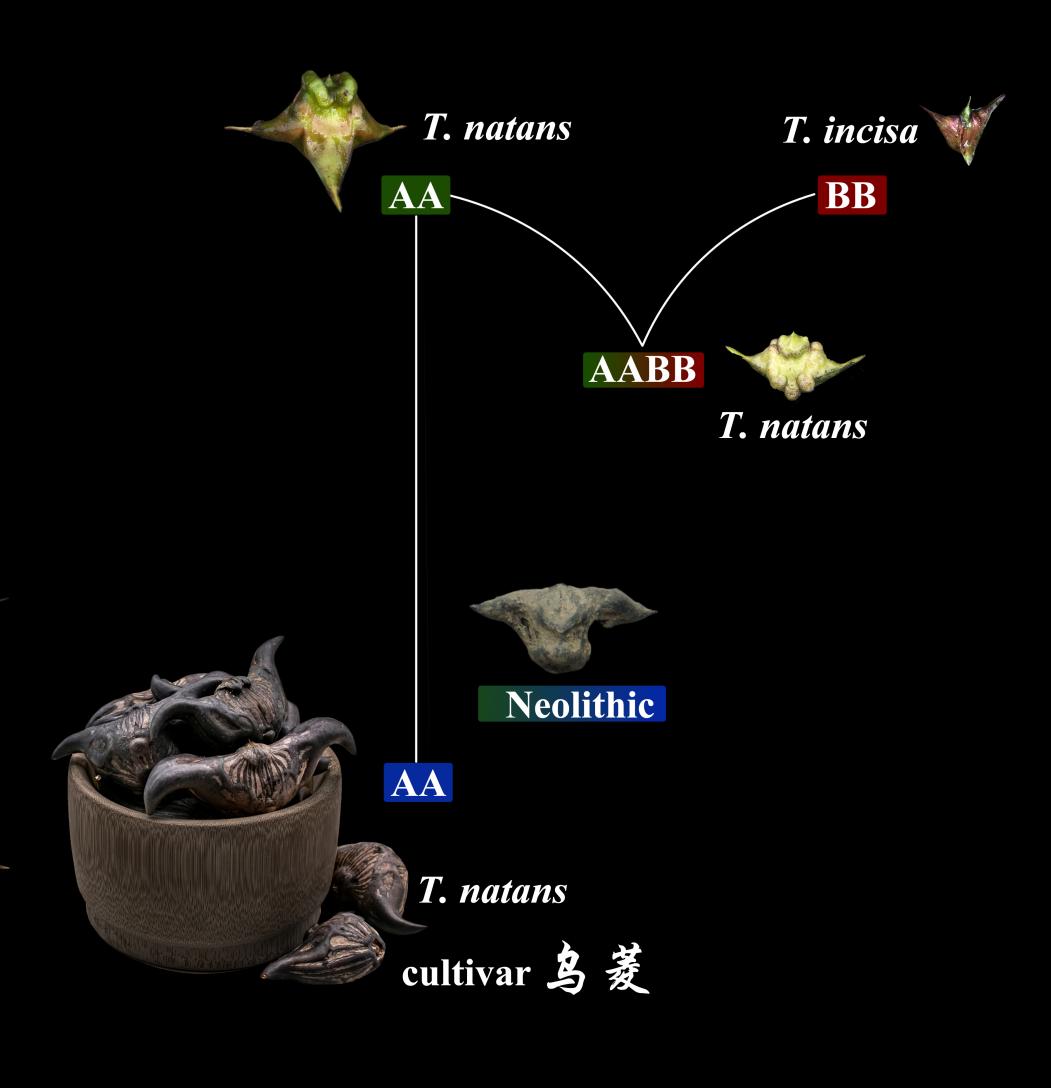


**Figure S1.** Schematic evolutionary histories for *Trapa* in China according to Lu *et al.*, 2022. The allotetraploid *T. natans* (4x, AABB) originated through hybridization between diploid *T. natans* (2x, AA) and *T. incisa* (2x, BB). Cultivated water caltrops were derived from diploid *T. natans* and likely first domesticated in the Yangtze Valley as early as 6,300 yr BP in China. Further improvement of the cultivar ‘Wuling’ took place *c*. 800 years ago.


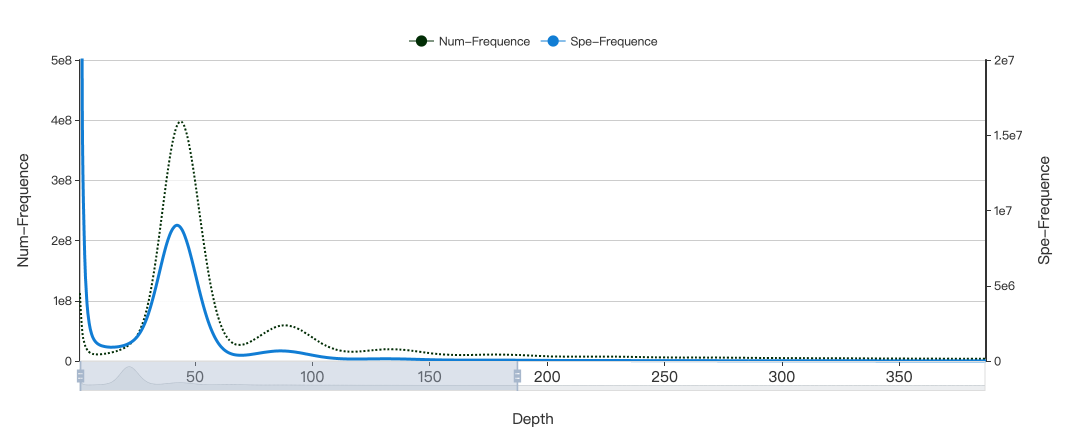


**Figure S2.** *K*-mer based genome size estimate for diploid *Trapa natans.* A total of 23,644,378,410 17-mers were detected with a peak depth of 44 and an estimated genome size of 529.46 Mb.


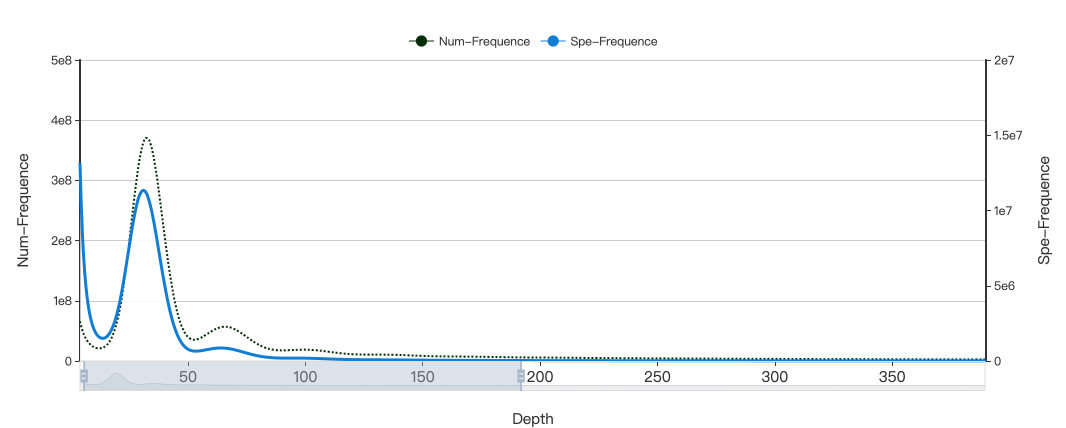


**Figure S3.** *K*-mer based genome size estimates for *Trapa incisa*. A total of 41,022,495,884 17-mers were detected with a peak depth of 80 and an estimated genome size of 503.46 Mb.


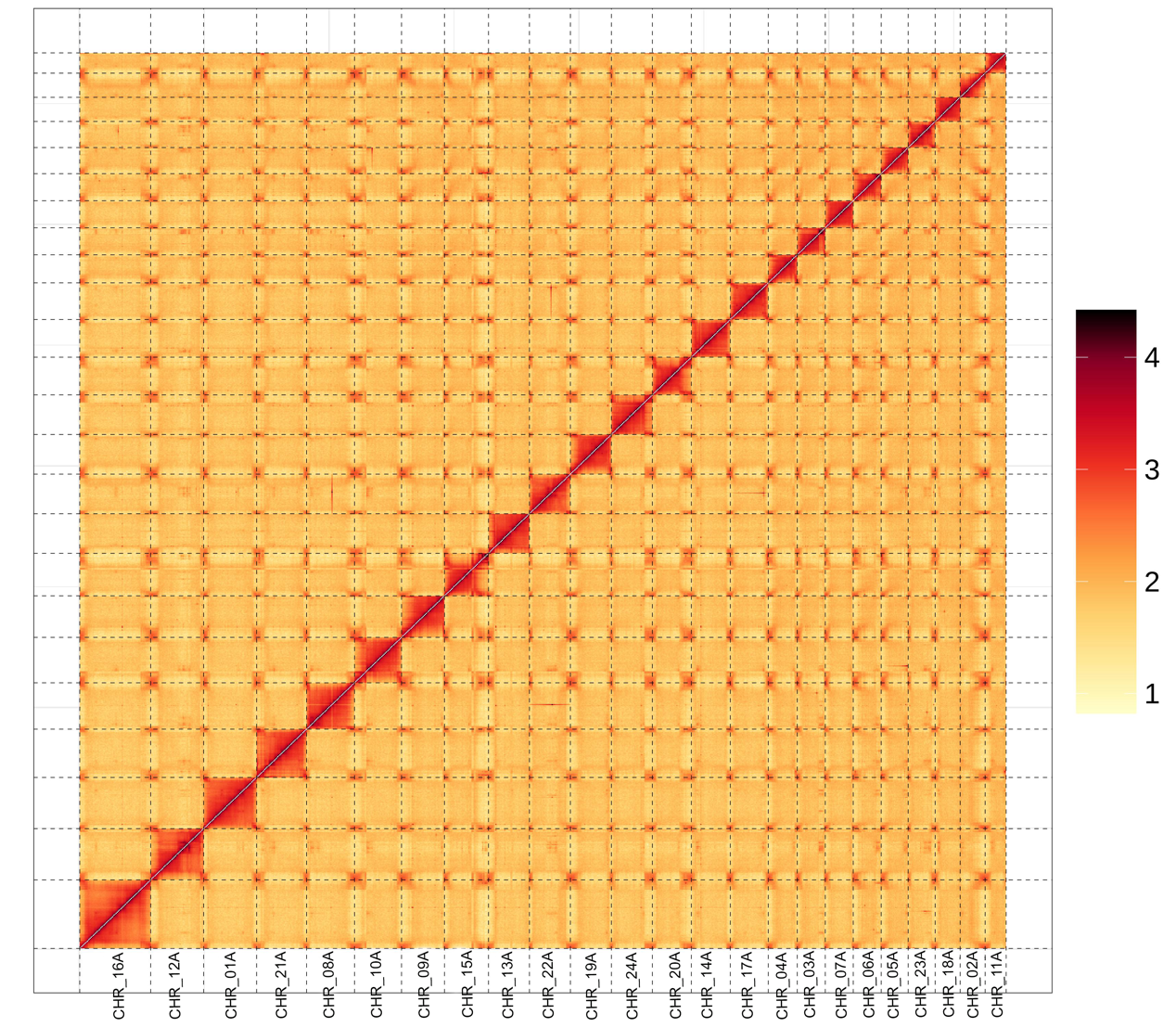


**Figure S4.** Genome-wide analysis of chromatin interactions between 24 chromosomes in diploid *Trapa natans* genome based on Hi-C data.

**
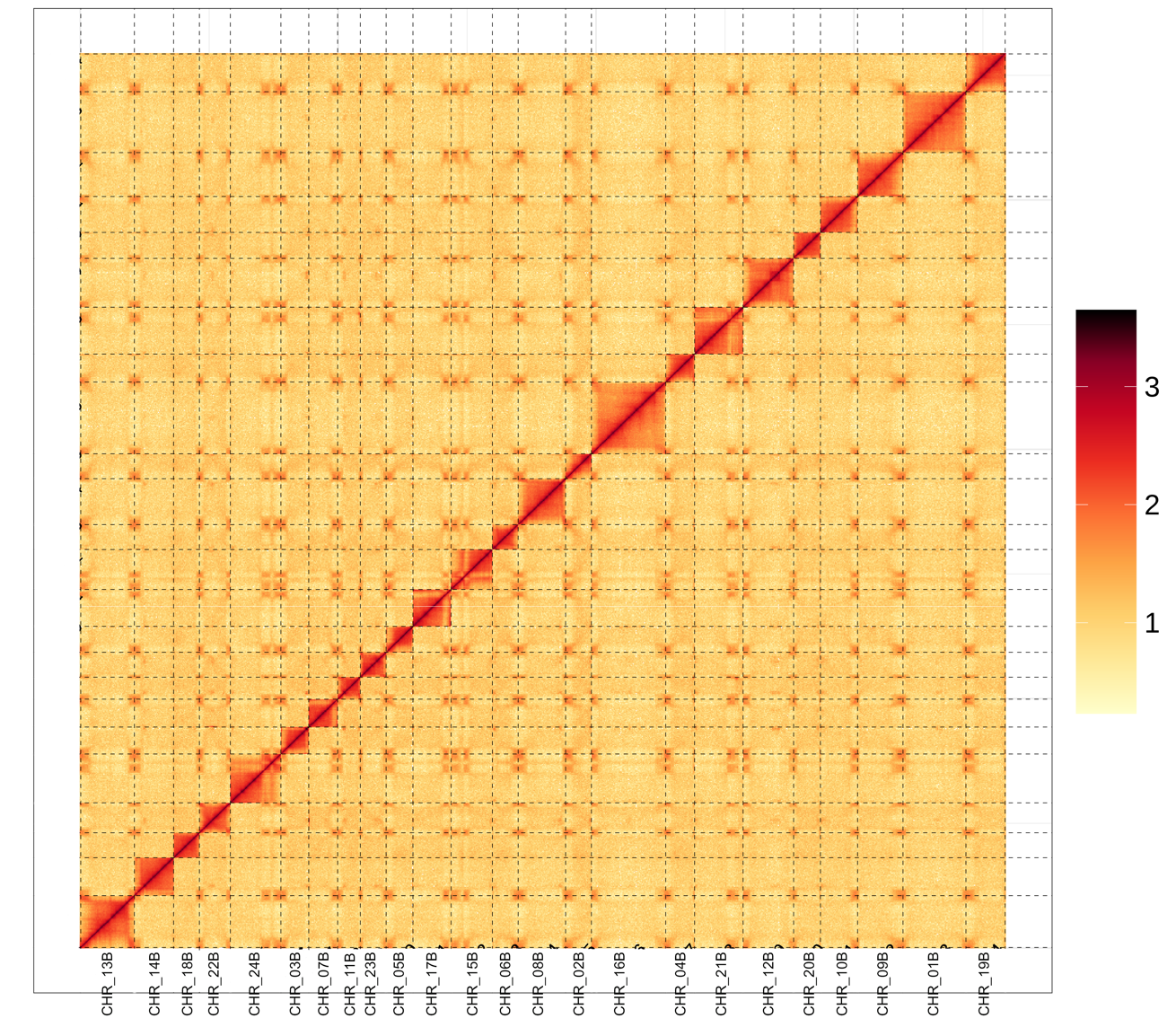
Figure S5.** Genome-wide analysis of chromatin interactions between 24 chromosomes in *Trapa incisa* genome based on Hi-C data.

**Figure S6.** The results of gene ontology (GO) enrichment analysis for core genes of *Trapa*. *P*-values of GO terms were calculated by Fisher’s Exact test.

**Figure S7.** The results of gene ontology (GO) enrichment analysis for dispensable genes of *Trapa*. *P*-values of GO terms were calculated by Fisher’s Exact test.


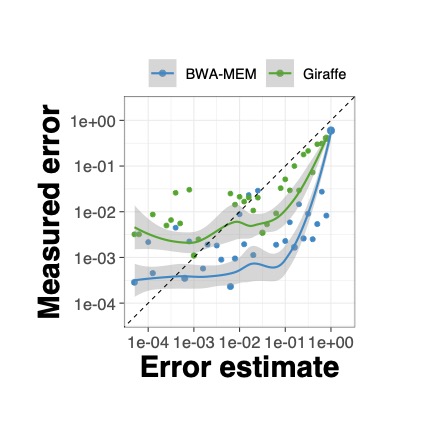


**Figure S8.** QQ plot for simulated read mapping with Illumina short reads for graph-based (green line) and linear genome (blue line) mapping approaches, respectively.


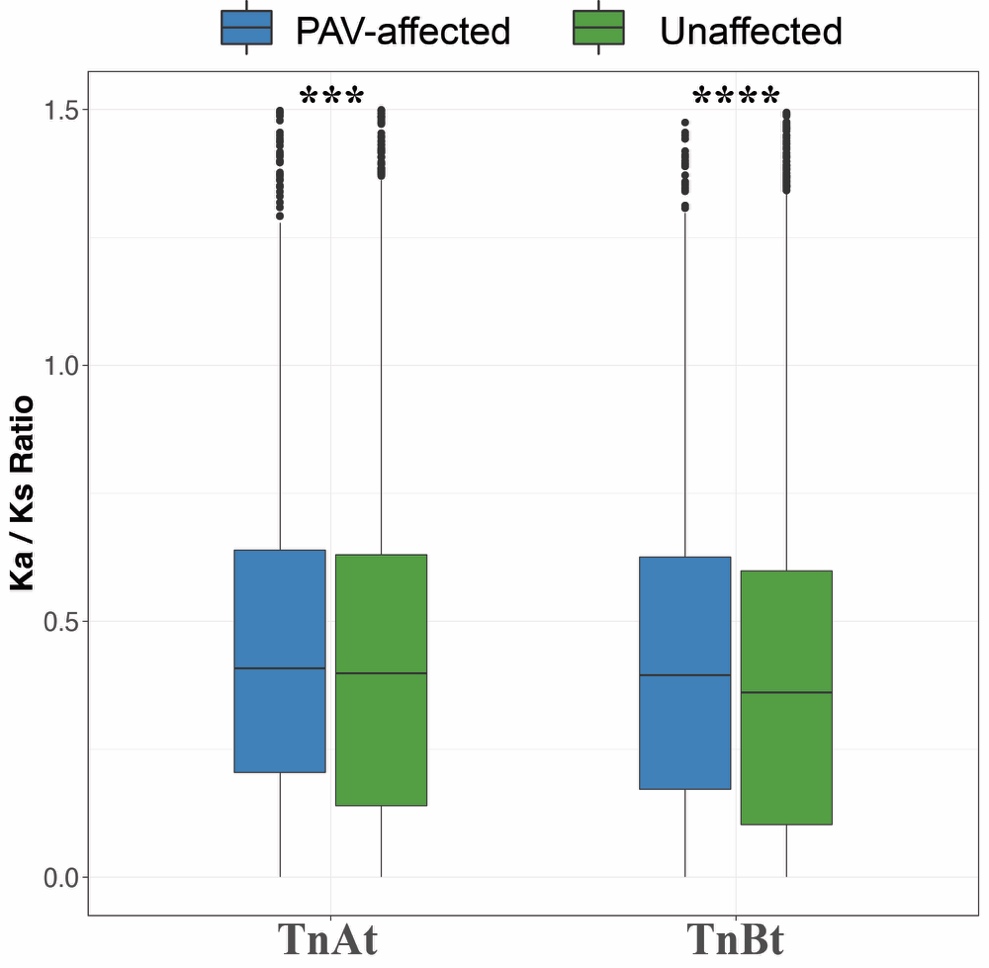


**Figure S9.** Comparisons of the *K*a/*K*s ratios between unaffected and PAV-affected genes in *TnAt* and *TnBt*.

**Figure S10.** Histograms of genome-wide expression of syntenic homeologous genes between *TnAt* and *TnBt* in four tissues, including (a) flowering bud (FB), (b) fertilized flower (FF), (c) juvenile fruit (JF), (d) and leaf (L).
